# Embedded Pork Bone Causing Esophageal Perforation and an Esophagus-Innominate Artery Fistula

**DOI:** 10.1155/2014/969862

**Published:** 2014-08-03

**Authors:** Andrew C. Berry, Peter V. Draganov, Brijesh B. Patel, Danny Avalos, Warren L. Reuther, Avinash Ravilla, Bruce B. Berry, Michael J. Monzel

**Affiliations:** ^1^Kansas City University of Medicine and Biosciences (KCUMB), 1750 E. Independence Avenue, Kansas City, MO 64106, USA; ^2^Department of Medicine and Advanced Therapeutic Endoscopy, College of Medicine, University of Florida, Gainesville, Florida, FL 32610, USA; ^3^Department of Internal Medicine, University of South Florida, Tampa, FL 33612, USA; ^4^Department of Internal Medicine, University of Miami Miller School of Medicine, Palm Beach Regional Campus, Atlantis, FL 33462, USA; ^5^Department of Radiology, West Palm Hospital, West Palm Beach, FL 33407, USA; ^6^Department of Internal Medicine, Wheaton Franciscan Healthcare, Milwaukee, WI 53215, USA; ^7^Digestive Disease Center of the Palm Beaches, Loxahatchee, FL 33470, USA

## Abstract

Chronically embedded foreign bodies can lead to perforations, mediastinitis, and abscess, amongst a host of other complications. A 20-year-old mentally challenged female presented with “something stuck in her throat,” severe dysphagia, and recurrent vomiting. Initial imaging was unremarkable; however, subsequent imaging and esophagogastroduodenoscopy two weeks later revealed an embedded pork bone. Surgery was performed to remove the bone and fix the subsequent esophageal perforation and esophagus-innominate artery fistula. This case helps reinforce the urgency in removing an ingested foreign body and the ramifications that may arise with chronically embedded foreign bodies.

## 1. Introduction

Foreign body ingestion is very common among the pediatric population and among adults who are mentally challenged or with psychiatric illnesses [1–3]. The esophagus is the most common anatomic location for a foreign body to impact [4]. Although most foreign bodies pass spontaneously, 10% to 20% of the cases require nonoperative intervention such as rigid endoscopy or flexible endoscopy. Less than 1% will require surgical intervention [5]. Serious life-threatening complications of foreign body ingestion do occur, but in less than 1% of the cases [6]. Of these complications, esophageal perforation carries a mortality rate that remains as high as 20% and can result in serious complications such as mediastinitis and retropharyngeal or parapharyngeal abscesses [6–8]. We describe the management of a chronically impacted esophageal foreign body not only causing an anterior and posterior perforation but also forming an esophagus to innominate artery fistula.

## 2. Case Report

**Figure 1:**
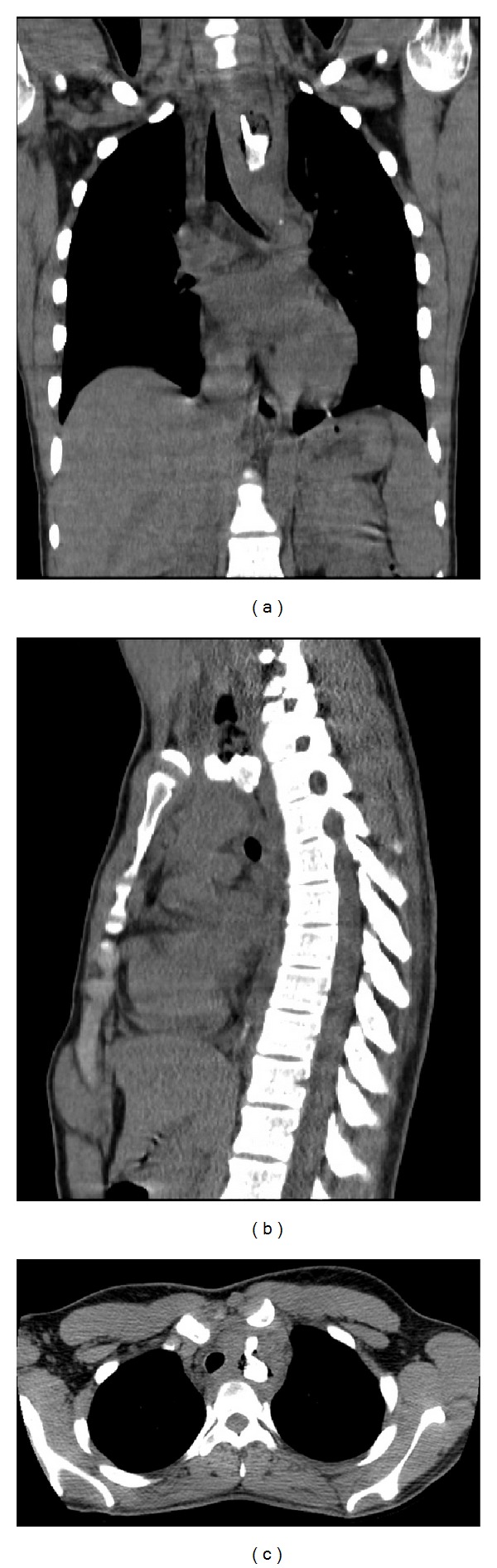
Computed tomography (CT) of the chest without contrast demonstrating a 3.2 cm (cranial/caudal) by 2.5 cm (anterior/posterior) Y-shaped foreign body within the anterior-superior mediastinum in the region of the esophagus. There is gas surrounding the mass. The foreign body has bone density and the gas is in part extraluminal. Edema is present in the circumference of the foreign body and is thought to shift the trachea from the left to the right, with compression of the airway. The soft tissue swelling is also evident surrounding the foreign body and esophagus, reflective of local inflammatory changes and hematoma secondary to the fistula connection between the esophagus and proximal innominate artery. (a) Coronal view. (b) Sagittal view. (c) Axial view.

A 20-year-old mentally challenged female complained of “something stuck in her throat,” severe dysphagia, and recurrent vomiting. She was seen two weeks priorly for possible pork bone ingestion; however, initial chest X-ray and soft tissue imaging of the neck were negative. Vital signs upon readmission were blood pressure of 106/80, heart rate of 131 beats per minute, respiratory rate of 20, temperature of 37.0°C, and an oxygen saturation of 98% on room air. Her physical exam was unremarkable. There was no evidence of subcutaneous crepitations. The patient's metabolic panel and complete blood count were within the normal range with the exception of hemoglobin 11.7 gm/dL. Computed tomography (CT) of the chest without IV or oral contrast showed a large foreign body (with air density most likely representing a bone) in the proximal esophagus at the level of the thoracic inlet, with compression of the airway and tracheal deviation to the right (Figure 1). No pneumomediastinum or subcutaneous emphysema was identified.

**Figure 2:**
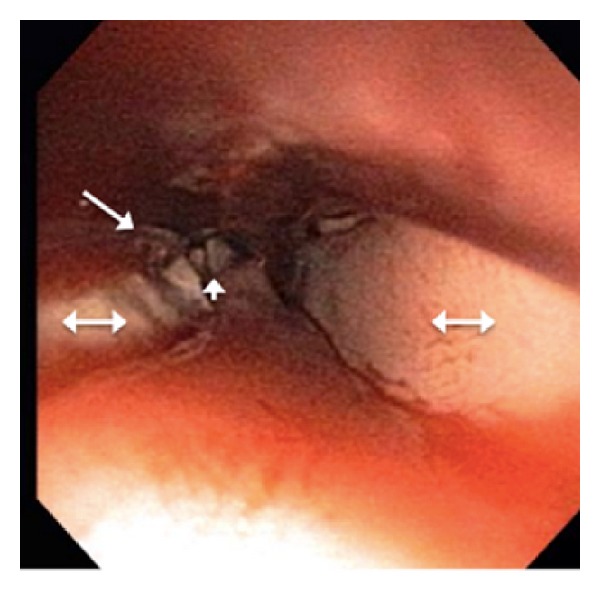
Esophagogastroduodenoscopy (EGD) showing a foreign body (arrowhead) embedded in the distal esophagus with ulcerations (single-sided arrow) in the surrounding mucosa, with subsequent surrounding edema (double-sided arrow).

Esophagogastroduodenoscopy (EGD) using a thin 5.5 mm diameter scope (Olympus, GIF XP180N) revealed a bone embedded into the esophagus (Figure 2). The scope could pass the embedded foreign body, but no attempt was made to remove it because it was difficult to ascertain the depth of penetration into the esophageal wall and the likelihood of an esophageal perforation. The patient was given antibiotics, thoracic surgery was consulted, and the patient was transferred to another hospital. Overnight the patient experienced coffee-ground emesis with occasional hematemesis.

At the next day, a rigid esophagoscopy under general anesthesia done in the operating room showed the upper esophagus filled with dark blood; as the scope was advanced new bright red blood was encountered. A surgical incision was made along the sternocleidomastoid muscle on the left side. Within the tracheoesophageal groove, a distended and blood-filled proximal esophagus was encountered. An esophageal incision was made and brisk blood came through the esophagus and the proximal innominate artery was identified. When pressure was relieved, bleeding was noted from the back wall of the innominate artery at its junction to the aortic arch.

Perforation of the esophagus extended into the back of the innominate artery. The artery was repaired with suture and the esophageal incision was extended to the perforation. A suction catheter helped grasp and excise the dislodged 3.2 cm × 2.5 cm Y-shaped bony fragment. The esophageal perforation and incision were closed with suture, a Jackson-Pratt drain was placed through the neck, sternotomy was closed, and a chest tube was inserted. The patient was critical throughout the procedure but was stable upon completion amid a 4-liter (L) blood loss. The patient made a gradual recovery and subsequent esophagram a week later showed no evidence of extravasation of contrast, obstruction, or strictures.

## 3. Discussion

Although the majority of foreign body ingestions can be managed conservatively and pass on their own through the GI tract, some cases require further endoscopic or even surgical management [5]. Foreign body esophageal impactions should be removed within 24 hours of the impaction to minimize the risk of perforation, mediastinitis, and abscess [9]. Our patient's initial CXR and soft tissue imaging of the neck were negative for any foreign body but identified two weeks later on CT of the neck and chest. Our case was refractory to all nonsurgical endoscopic intervention. It ultimately required a sternotomy to facilitate removal. The pork bone became embedded within the esophageal wall over a two-week period leading to mucosal edema and narrowing of the passageway. The chronically embedded foreign body led to an anterior and posterior esophageal perforation and an esophagus-innominate artery fistula.

Proper management of an esophageal foreign body removal depends on the size, location, and degree of tissue penetration. If plain films do not successfully locate a foreign body, an esophagram with barium or gastrografin can be performed. However, an esophagram is not recommended due to its increased risk of aspiration [10] and by its high reported false-positive and false-negative rates [11]. CT has been shown to be most sensitive for localizing esophageal foreign bodies and provides superior benefit to other modalities in locating additional complications, such as esophageal perforations [12]. If the foreign body does not pass quickly on its own, endoscopic management is warranted. This typically involves endoscopic rat-tooth forceps. In selected cases a dual-channel endoscope may be more advantageous than a standard flexible endoscope because of its greater diameter and ability to be used with a balloon catheter to minimize mucosal damage by expanding the esophagus [13]. If severe risk of bleeding or perforation is evident, as in our case, no further endoscopic evaluation should occur and urgent surgical evaluation is warranted to remove the foreign body and manage any bleeding or perforations. Had the foreign body been removed endoscopically and esophageal perforation was later noted, endoscopically placed removable esophageal stents, combined with aggressive conservative therapy, remain the standard of care [14].

Our case is unique because the foreign body remained embedded in the esophagus for an extended period of time making its removal an arduous task amid an esophagus-innominate artery fistula and an esophageal perforation. This case helps reinforce the urgency in removing an ingested foreign body and the ramifications that may arise with chronically embedded foreign bodies. Proper imaging work-up and management must be swiftly initiated to help identify any foreign bodies and any associated tissue changes.
